# Effects of tongue-hold swallows on suprahyoid muscle activation according to the relative tongue protrusion length: a preliminary study

**DOI:** 10.1186/s40064-016-2799-8

**Published:** 2016-07-21

**Authors:** Jong-Chi Oh

**Affiliations:** Department of Occupational Therapy, Cheongju University, 298 Daesung-ro, Cheongwon-gu, Cheongju, Chungcheongbuk-do 28503 Republic of Korea

**Keywords:** Deglutition, Deglutition disorders, Electromyography, Overload, Tongue

## Abstract

Tongue-hold swallow (THS) is a therapeutic maneuver used to increase the posterior pharyngeal wall motion during swallowing. This maneuver has also been reported to result in increased activation of the suprahyoid muscles. The hypothesis of this study was that the degree of suprahyoid muscle activation would depend on the tongue protrusion-length. The aim of this study was to investigate the activation levels of the suprahyoid muscles by surface electromyography (sEMG) while performing the THS maneuver at three tongue-protrusion lengths. Suprahyoid muscle activity during THSs was recorded in 25 adult volunteers (17 women and 8 men; age range 20–38 years). To record the activity of the suprahyoid muscles while the participants performed the maneuver, surface wireless EMG electrodes separated by a distance of 1 cm were placed on the skin on both sides of the midline under the chin. Each activity was recorded three times. Data analysis was performed by repeated-measures analysis of variance. Our results revealed that participants exhibited greater electrical activity during THS with 2/3rd or maximal tongue protrusion as compared to THS with 1/3rd tongue protrusion (p ≤ 0.001). To maximize the therapeutic effect of the THS maneuver, it is advised to protrude the tongue maximally as long as swallowing is possible.

## Background

Tongue-hold swallow (THS) is a therapeutic maneuver that helps increase the posterior pharyngeal wall motion during swallowing (Fujiu and Logemann 1996; Lazarus et al. 2002). It has been surmised that this technique can improve the contact between the tongue base and the pharyngeal wall by increasing the pharyngeal wall contraction during swallowing (Fujiu-Kurachi et al. 2014). Fujiu and Logemann (Fujiu and Logemann 1996) reported that young healthy male adults exhibited a greater increase in anterior bulge of the posterior pharyngeal wall during THS as compared to their swallows without this maneuver. Lazarus et al. (Lazarus et al. 2002) identified that patients with head and neck cancer exhibited a significantly greater increase in duration of contact and pressure of the base of tongue to the posterior pharyngeal wall during THS as compared to their swallows without this maneuver. In addition, THS has been known to result in an increase in suprahyoid muscle contraction. Hammer et al. (Hammer et al. 2014) compared the effects of three variations of THS—saliva swallowing without any maneuver, with the tip of the tongue at the lip, and with the universal tongue-hold maneuver—using hook-wire intramuscular electromyography. In their study, healthy subjects exhibited a greater increase in the magnitude and duration of suprahyoid, genioglossus, and superior pharyngeal constrictor muscle activity during saliva swallowing with the tip of the tongue at the lip and with the universal tongue hold maneuver as compared to saliva swallowing without any maneuver.

The effect of THS likely depends on the overload principle, similar to that with general strengthening exercises. The principle of overload holds that, in order to increase the force-generating ability of a muscle, that muscle must be taxed beyond its current capacity to respond. That is, it must be exposed to a load greater than it is typically exposed to on a daily basis (Wheeler-Hegland et al. 2008). In THS, applied resistance on the pharyngeal wall corresponds to the restricted tongue movements during pharyngeal swallowing (Fujiu and Logemann 1996; Fujiu-Kurachi et al. 2014).

In an original THS study (Fujiu and Logemann 1996), participants were asked to maximally but comfortably protrude their tongue, holding it between the central incisors. In this instruction, “maximally but comfortably” could be interpreted subjectively, thus causing variations in tongue-hold lengths among patients, or within the same patient at different attempts. Therefore, it appears that a more objective criterion is needed for THS. Given that each person has a different tongue length (Fujiu-Kurachi et al. 2014), uniform protrusion of the tongue during THS, regardless of individual tongue length, would cause different effects, contrary to the expectations of many clinicians. It is hypothesized that the greater the tongue protrusion in proportion to individual tongue length, the greater the effort required for further compensation of restricted tongue movement. This increased effort can be measured by suprahyoid muscle surface electromyography (sEMG).

Fujiu-Kurachi et al. (2014) examined the changes in intraoral pressure during THS relative to different degrees of tongue protrusion—dry swallow with no tongue protrusion and THS with 1- and 2-cm tongue protrusion—in 18 young healthy adults. The participants exhibited interesting results. Two types of changes were identified in the maximal magnitude of tongue pressure for posterior intraoral pressure during THS; while eight subjects exhibited an increase in maximal magnitude, along with an increase in the extent of tongue protrusion (increase group), nine exhibited a decrease in maximal magnitude with the increase in extent of protrusion (decrease group). It was found that all eight subjects of the increase group had maximal tongue lengths >32 mm, whereas eight of the nine subjects of the decrease group exhibited maximal tongue lengths <32 mm. The authors suggested the possibility that the tongue of an individual with a greater range of protrusion has sufficient functional reserve for the required tongue movement, even with the tongue-holding maneuver, and is capable of compensating for the constrained movement of the anterior tongue. These results imply that the THS maneuver might require differential application. Therefore, the purpose of this study was to examine the changes in suprahyoid muscle activation during THS relative to different degrees of tongue protrusion in healthy adults.

## Methods

### Participants

This study included 25 volunteers (17 women and 8 men; mean age 24.0 ± 6.5 years; range 20–38 years) without a reported history of speech or swallowing deficits. All participants passed a swallowing screening test (Gugging Swallowing Screen) (Trapl et al. 2007) administered by a skilled occupational therapist who majored in dysphagia therapy. None of the participants reported drug use that could affect swallowing or neurological functions or having engaged in any type of swallowing-related strength training program for at least 1 year prior to this study. Before commencement of the study, all participants received a complete explanation of the purpose, risks, and procedures of the investigation, and provided written informed consent. Procedures were performed in accordance with the ethical standards of the committee on human experimentation at the institution at which the work was conducted, and this study was approved by the Institutional Review Board (201410-DW-038).

### Maximal tongue-protrusion length (TPL)

The distance between the tip of the tongue during maximum protrusion and the upper incisors was determined to be the maximal TPL (Fujiu-Kurachi et al. 2014). The maximal TPL was measured three times in each participant, and the mean value was considered the maximal TPL for that participant. To avoid the effect of variations in width and tonus of the tongue among individuals on the swallowing physiology, all participants were instructed to protrude their tongue maximally and maintain the tongue flat.

### Experimental procedure

Each participant performed three tasks, each of which was repeated three times: (1) THS with 1/3rd tongue protrusion, (2) THS with 2/3rd tongue protrusion, and (3) THS with maximal tongue protrusion. The participants were instructed to protrude the tongue to the predetermined length and maintain their tongue flat to avoid the effect of variations in width and tonus of the tongue among individuals on the swallowing physiology. The examiner evaluated the TPL using a colorless and transparent plastic ruler, measuring the distance (cm) between the upper incisors and the tip of the protruded tongue. The participants were allowed enough practice time to familiarize themselves with the three swallowing tasks prior to measurement; after practicing, they were allowed to rest for more than 15 min before measurements (Fujiu-Kurachi et al. 2014). Measurements were preceded by a familiarization session in order to exclude the effects of the learning curve and improve the reliability of the measurements. The measurements were then repeated three times, with a 120-s rest period between trials. The order of the tasks was randomized. A detailed description of the implementation of the tasks is included below.

### THS under three conditions

(1) THS with 1/3rd tongue protrusion (protrude the tongue to as much as 1/3rd of the premeasured maximal TPL, holding it between the incisors), (2) THS with 2/3rd tongue protrusion (protrude the tongue to as much as 2/3 of the premeasured maximal TPL, holding it between the incisors), (3) THS with maximal tongue protrusion (protrude the tongue as much as the extreme length, holding it between the incisors).

The participants swallowed saliva normally under each condition.

### Electrophysiological evaluation

For recording the suprahyoid muscle complex (mylohyoid, geniohyoid, and anterior digastric muscles) activity, surface wireless EMG electrodes were placed at distance of 1 cm on the skin on both sides of the midline under the chin (Beckmann et al. 2015). A two-channel electromyogram device (BTS FreeEMG 1000; BTS Bioengineering, Italy) was used to record the muscle activity. During the examination, participants were instructed to sit on a chair and hold their trunk in a neutral upright position. Prior to measurement under experimental conditions, the submental skin of each participant was cleansed with an alcohol wipe and allowed to dry for approximately 30 s. The signals were filtered (10–500 Hz), amplified, rectified, and then integrated. The recorded surface electromyography (sEMG) data were analyzed offline. The onset and offset signals representing the effort by the participant for each task were identified, and the signals in-between were analyzed to obtain the peak values (peak amplitude) for each participant. The onset on swallowing was defined as the point at which the signal activity of the electromyogram exceeded 2-standard deviation (SD) from the mean value at baseline that led to the swallowing event. The offset on swallowing was defined as the point at which the signal activity was below 2-SD from the mean value at baseline (Sakuma and Kida 2010). A total of nine sEMG measures were obtained for each participant. The mean values of the three trials for each task were used for the analysis of peak value.

### Statistical analysis

The experimental design of this study was within-subject, with repeated measures of the task conditions. Differences in peak suprahyoid muscle activation during three tasks were evaluated by comparing the peak sEMG measurements of the participants by repeated-measures analysis of variance. When one of the tasks was identified as having a significant effect, as determined by the F value, post hoc mean comparisons were analyzed with pairwise comparisons in order to identify the task responsible for the significant difference. Significance level was set at *p* < 0.025 (Bonferroni’s correction for multiple comparisons: 0.05/2 = 0.025). All data were analyzed using the SPSS version 18.0 software.

## Results

All 25 participants successfully completed the study. The mean values of maximal, 1/3rd, and 2/3rd TPL were 28.8 ± 3.74 mm (range 23–35 mm), 9.6 ± 1.25 mm (range 6.7–11.7 mm), 19.2 ± 2.49 mm (range 13.3–23.3 mm), respectively.

### Comparison of peak suprahyoid muscle activation levels according to the length of tongue-protrusion during normal swallowing

**Fig. 1:**
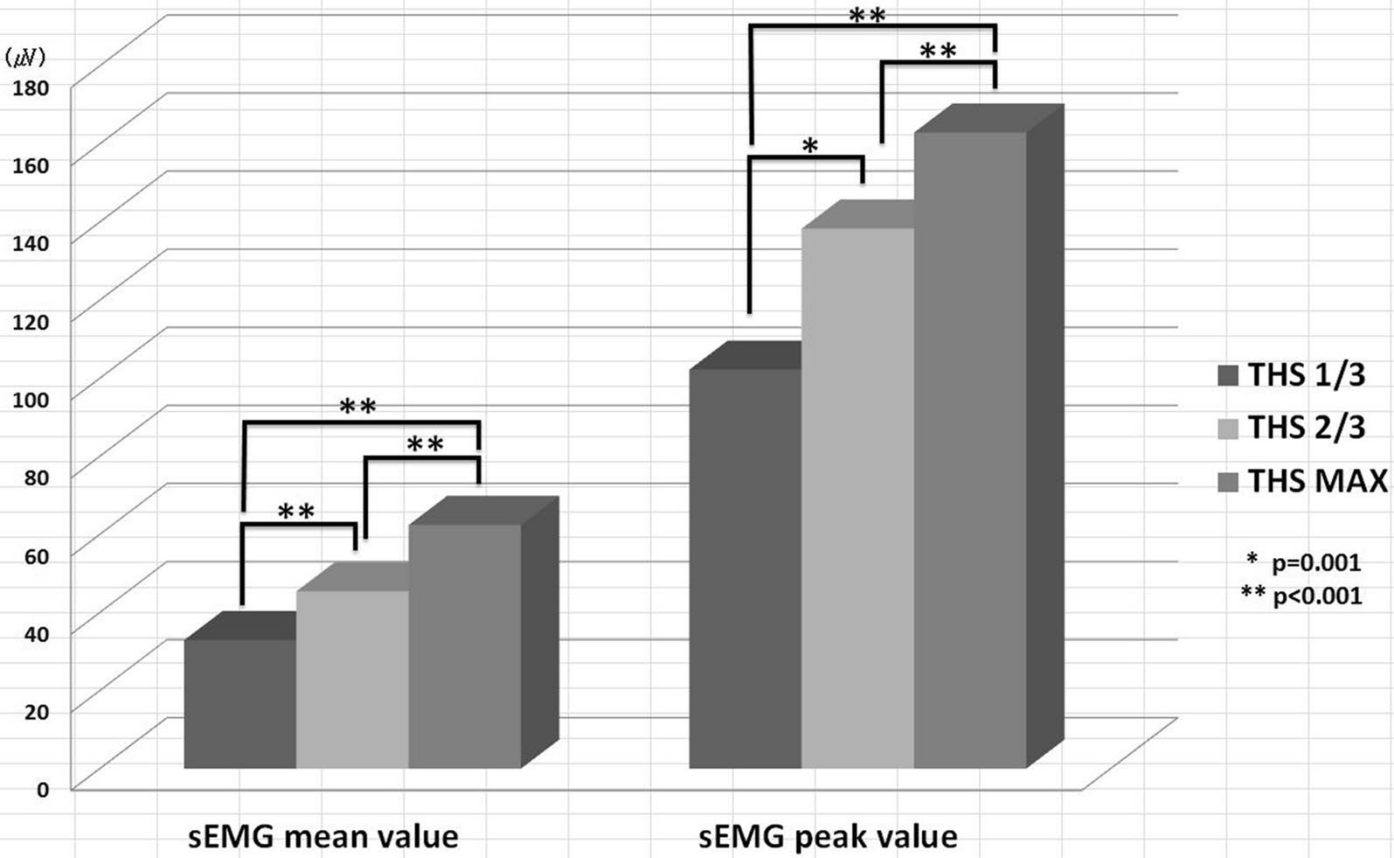
Comparison of mean and peak submental sEMG values during 3 tasks

Table 1 presents the comparison of peak sEMG values of each tasks with those obtained during THS with 1/3rd tongue protrusion. The results of evaluation of peak suprahyoid muscle activation values indicated that the suprahyoid muscles exhibited greater electrical activity during THS with 2/3rd (p = 0.001) and maximal tongue protrusion (p < 0.001) as compared with to THS with 1/3rd tongue protrusion. In addition, the increase in peak suprahyoid muscle activation during THS with maximal protrusion was significantly greater compared to that during THS with 2/3rd protrusion (p < 0.001) (Fig. 1).

**Table 1:** Maximum sEMG values of tasks compared to THS—1/3

Task	sEMG peak value ± SD	*p* value
THS—1/3 tongue protrusion	102.2 ± 37.6	
THS—2/3 tongue protrusion	138.3 ± 64.7*	0.001
THS—Max tongue protrusion	162.9 ± 57.5*	<0.001

*THS* tongue-hold swallow, *Max* Maximum

* Significant difference vs. THS—1/3 (p < 0.025)

## Discussion

The aim of the present study was to evaluate the changes in suprahyoid muscle activation across three conditions during normal swallowing with tongue holding. Previous studies have indicated that THS induces greater intraoral pressure and suprahyoid muscle activation as compared to normal saliva swallowing without tongue holding (Fujiu-Kurachi et al. 2014; Hammer et al. 2014). The results of the present study further indicated that THS with 2/3rd and maximal protrusion induced significantly higher suprahyoid muscle activation as compared to THS with 1/3rd protrusion. In addition, the increase in peak suprahyoid muscle activation during THS with maximal protrusion was found to be significantly higher as compared to that during THS with 2/3rd protrusion (Fig. 1). These results suggest that the greater the protrusion of the tongue, the greater the suprahyoid muscle activation during swallowing.

The lengths of tongue protrusion evaluated in the present study were 1/3rd, 2/3rd, and maximum length of the maximal TPL of each participant; the mean values of TPL were 9.6, 19.2, and 28.8 mm, respectively. These values are mostly similar to those reported by Fujiu-Kurachi et al. (10, 20 mm) (Fujiu-Kurachi et al. 2014). However, the range of TPL for 1/3rd 2/3rd protrusion were 6.7–11.7 and 13.3–23.3 mm, respectively, which indicates the considerably wide variation in maximal tongue protrusion length (TPL) among individuals. In addition, the range of maximal TPL observed in the present study (23–35 mm) differs from that reported by Fujiu-Kurachi et al. (24–48 mm). In the latter study, the greater tongue pressure observed among participants with longer tongue (>32 mm) during 2-cm THS compared to that observed during 1-cm THS might have been a result of the contribution of the movable residual tongue base in order to compensate for the anchored anterior tongue and produce sufficient swallowing pressure. This phenomenon could be a barrier for effecting posterior pharyngeal wall movements, which is the original goal of THS. In the present study, suprahyoid muscle activations during maximum and 2/3rd protrusion of the tongue were far greater compared to that during 1/3rd protrusion under normal swallowing conditions. Therefore, THS with 2/3rd or maximum tongue protrusion might result in better outcomes than THS with 1/3rd protrusion. The THS maneuver is a strengthening exercise prescribed to patients who exhibit decreased pharyngeal constriction. To maximize the therapeutic effect of THS, it is advisable to protrude the tongue maximally as long as swallowing is possible. Clinically, several patients cannot perform this maneuver because the anchorage of the tongue makes swallowing very difficult. Therefore, to impose a realistic maximum load, it appears more feasible to start with an appropriate TPL according to the individual maximal TPL that allows swallowing with a surmountable resistance rather than wildly protruding the tongue maximally or employing a fixed TPL irrespective of individual tongue length. The length of protrusion could be increased progressively according to the progression of the patient.

Three limitations of the present study need to be acknowledged. First, the dependent variable evaluated in this study was suprahyoid muscle activation measured by sEMG. These surface recordings serve as an estimate of the combined activity of the muscles of the floor of the mouth (Palmer et al. 1999). Although this could possibly reflect pharyngeal musculature activation according to THS conditions, actual pharyngeal wall movement or contraction cannot be measured directly. Therefore, further studies using videofluoroscopy or oropharyngeal manometry should be performed for the evaluation of these changes. Second, this study measured immediate suprahyoid muscle activation at three different TPLs. To compare the therapeutic effects of these three TPLs in terms of strengthening the suprahyoid muscles, further studies involving the application of this exercise as part of long-term programs are required. Third, this study was conducted with healthy young adults without dysphagia. Further studies including older subjects or patients with dysphagia who shows pharyngeal weakness could increase our understanding of the differences in therapeutic outcomes of THS according to the TPL.

## Conclusions

In summary, the results of the present study indicate that the greater the tongue protrusion length, the greater the activation of the suprahyoid muscles while swallowing. Therefore, to maximize the therapeutic effects of the THS maneuver, it is advised to protrude the tongue maximally as long as swallowing is possible.
